# Rationale and Design of the Brigham Cohort for psoriasis and psoriatic arthritis registry (COPPAR)

**DOI:** 10.1186/s12895-017-0063-8

**Published:** 2017-08-16

**Authors:** Maria Schneeweiss, Joseph F. Merola, Elizabeth W. Karlson, Daniel H. Solomon

**Affiliations:** 1 Division of Rheumatology, Department of Medicine of the Brigham and Women’s Hospital, Harvard Medical School, 75 Francis Street, Boston, MA 02115 USA; 2 Department of Dermatology of the Brigham and Women’s Hospital, Harvard Medical School, Boston, USA; 3 Division of Pharmacoepidemiology, Department of Medicine of the Brigham and Women’s Hospital, Harvard Medical School, 75 Francis Street, Boston, MA 02115 USA

**Keywords:** Psoriasis, Psoriatic arthritis, Disease registry, Biomarkers, Electronic health record

## Abstract

**Background:**

Psoriasis (PsO) and psoriatic arthritis (PsA) are related conditions with poorly defined transition among them, risk factors for progression, complex treatment algorithms, and biomarkers for treatment response and long-term outcomes. We describe the development of a PsO/PsA registry at an academic medical center.

**Methods:**

We developed a single-center PsO/PsA longitudinal disease registry including biorepository that captures relevant disease markers and treatment choices in a circumscribed population with a defined catchment area. We searched the electronic medical record for patients with visits in the last year for PsO or PsA. They formed the potentially eligible registry population. Baseline patient and provider questionnaires were developed using standardized measures, including demographics, comorbidities, medications, specific disease characteristics, functional status, quality of life, mental health, and resource use. An abbreviated set of items was collected every six month and at visits with treatment changes or disease flares. Biospecimens included blood (serum, plasma, DNA, RNA) and skin biopsy samples, with repeat collections of serum and plasma. Data from the EMR to augment the registry questionnaires are available on all patients.

**Discussion:**

Searching the Brigham EMR system from 2013 through 2014, we found 1694 patients with PsO and 1028 with PsA. Their mean age was 55 years and 53% were female. Of these 17% had diabetes, 38% hyperlipidemia, and 45% hypertension. The median BMI was 29.6. PsA patients used more systemic prednisone, MTX, and TNF alpha inhibitors (47%, 60%, and 66%) compared to PsO patients (28%, 20% and 21%). We have collected plasma in 410 patients, DNA/RNA in 453 patients. In conclusion, we have developed a PsO/PsA registry to better define longitudinal disease characteristics, perform biomarker studies, and examine treatment trends.

## What is the most significant finding of this registry?

This paper describes the structure and functioning of a new psoriasis and psoriatic arthritis disease registry at a major medical center. Early results showed that registry participants were representative for the population of the catchment area.

## What does it mean for dermatologists and their patients

The psoriasis and psoriatic arthritis disease registry will allow new research on the natural progression of disease, typical treatment pathways and the effectiveness of new treatment on clinical and patient-reported outcomes.

## Learning points

1. There is a wave of new therapeutic options for chronic skin diseases, particularly complex conditions like psoriatic arthritis
2. In order to better understand how new therapies work in routine care of patients with psoriatic arthritis detailed clinical information is necessary
3. We established a new psoriatic arthritis registry that is embedded in an academic center with a large population with psoriatic arthritis and substantial clinical expertise
4. The new registry will initially include more than 1000 patients with psoriatic arthritis that are longitudinally followed
5. The registry includes validated instruments for physician and patient reported severity and outcome measures plus biomarker and genetic information to study the effectiveness of new therapies

## Background

Disease registries can fill an important gap for improving the understanding of chronic diseases with various treatment strategies. They capture patients in routine care “real-world” settings and collect information on clinical details, biologic materials, patient reported outcomes, and clinical treatment pathways [1]. These types of observational data stand in contrast to most randomized controlled trials that describe highly selected populations. Registries serve complementary roles to studies based on electronic health records with incomplete outcomes information and sparse biospecimens. For patients with psoriasis (PsO) and psoriatic arthritis (PsA) there are few disease registries in Europe [2, 3] and the Americas [4, 5]. Some have clearly defined catchment areas and can link to existing national registries [3], others are more geographically diverse [2]. Despite the existing registries, a recent review paper concludes with a call for more PsA registries with systematic capture of patient reported outcomes and bio-specimens [6]. Furthermore, the development of systemic arthritis in patients with PsO remains a challenging topic of research; thus, a combined PsO and PsA registry provides important research opportunities.

In considering the development of a combined PsO/PsA registry, we considered several research opportunities and challenges. First, disease registry information is critically important to understand the natural course of PsO and PsA utilizing current treatment paradigms. Treatment options for both conditions have rapidly expanded over the last decade and will continue to broaden. A detailed understanding of how and when patients and their providers decide to transition between treatments is important for improving care; it also allow for comparative effectiveness studies to assess real-world benefits and toxicities. Second, around one-third of patients with PsO develop systemic inflammatory arthritis consistent with PsA [7]. The predictors of PsO to PsA transition include genetic, environmental, and physical examination findings [7, 8], but a deeper understanding of the biology of this transition might open up preventive strategies, where there are currently none [9]. Third, registries allow for capture of varied types of data but additional information on comorbid conditions and medication use can be gleaned from linkage between registry data with electronic medical record and health care claims. Finally, PsO has many different clinical phenotypes, including nail disease, scalp, palmo-plantar, pustular, genital, and inverse (intertriginous). Scalp, nail and inverse psoriasis sub-types are common and are associated with an increased risk of PsA, however response to treatments of these subsets has not been well characterized.

Herein, we describe the development of a single-center PsO/PsA longitudinal disease registry including biorepository that captures these elements in a circumscribed population with a defined catchment area. The registry is named COPPAR, COhort for Psoriasis and Psoriatic Arthritis Registry, of the Brigham and Women’s Hospital in Boston. The specific goals of the registry are to determine biologic, clinical and environmental predictors of PsA among patients initially presenting with PsO; to identify predictors of treatment response and failure and characterize treatment transitions; to quantify health services utilization and quality of life of patients with PsO and PsA; and to assess relevant subgroups of patients with specific phenotypes, including non-plaque disease (e.g., nail disease, palmar-plantar, genital, inverse).

## Methods

### Patients eligible and included in the registry

The patients eligible for the COPPAR registry have been seen for PsO or PsA at Brigham and Women’s Hospital, a large academic medical center in Boston. The hospital’s Center for Skin and Related Musculoskeletal Diseases (SARM), a specialist clinic treating patients with concomitant systemic rheumatic and skin diseases [10], has an extensive referral network within the catchment area making the patient population representative of a Northeastern metropolitan population. Patients that might qualify for the registry are identified by systematically screening the electronic medical record (EMR) system of the hospital using an algorithm previously found to be highly predictive of psoriasis [11, 12]. Since the identified potentially eligible subjects will all have their diagnoses confirmed before entering the registry, we simplified the search algorithm to include patients with at least three diagnoses of PsO (ICD-9-CM code 696.1 or ICD-10 code L40.0) or at least three diagnoses of PsA (ICD-9-CM code 696.0 or ICD-10 code L40.5) who also have had a visit to the hospital from 2013 through 2014 for either diagnosis [13, 14].

Using this search strategy, we identify 2484 potentially eligible subjects who have now been invited to participate in the registry. During the baseline visit the treating physician confirms and records the clinical diagnosis using the CASPAR criteria for PsA [15] or the dermatologist-defined diagnosis of PsO [14, 16]. Patients not meeting these criteria will be excluded from participating in the registry.

### Questionnaire development

The registry questionnaires were developed based on an extensive review of validated instruments. Broad areas of interest in the registry include demographics, comorbidities, medications, severity and activity of PsO/PsA, functional status, quality of life, physical activity, mental health [17], health resource utilization, and physical examination [18]. We consulted with disease experts, outcomes researchers and experts in questionnaires to confirm the final instrument selection (Table 1).

**Table 1:** Key items recorded by the COPPAR registry

Variables	Physician Assessed	Patient Assessed
Socio-demographic		
Demographics		✔
Age		✔
Education		✔
Ethnicity		✔
Psoriasis (PsO)/Psoriatic Arthritis (PsA)		
First PsO or PsA Diagnosis		✔
Family History of PsO and/or PsA	✔	✔
CASPAR	✔	
Physician Global (VAS)	✔	
General Health Features		
Co-morbidities / Drug Toxicities	✔	✔
Cardiovascular Risk Factors	✔	✔
Infections/Opportunistic Infections	✔	✔
Surgical History		✔
Smoking Status		✔
Alcohol Consumption		✔
Mental health (CESD)		✔
Health Care Utilization		✔
Medications		
Current	✔	✔
Past	✔	✔
Changes	✔	✔
Start	✔	✔
Stop/reason	✔	✔
Change/reason	✔	✔
Peripheral Joint Assessment		
66/68 tender/swollen joint count	✔	
Patient swollen joint assessment (VAS)		✔
Skin Assessment		
Psoriasis Area Severity Index (PASI)	✔	
Body Surface Area (BSA)	✔	
Physician Static Global Assessment (sPGA)	✔	
Pain		
Patient tender joint assessment (VAS)	✔	✔
Patient Global		
VAS		✔
Pain and Function		
Multi-Dimensional Health Assessment Questionnaire (MDHAQ)		✔
Health-related quality of life		
European Quality of Life assessment (EuroQol)		✔
Dermatology Life Quality Index (DLQI)		✔
Psoriatic Arthritis Quality of Life (PsAQoL)		✔
Enthesitis		
LEEDS Enthesitis Index (LEI)	✔	
Dactylitis		
Absent/Present	✔	
Spinal assessment		
Absent/ Present	✔	
Bath Ankylosing Spondylitis Disease Activity Index (BASDAI)		✔
Psoriasis Assessment		
CAPP – Plaque Psoriasis	✔	✔
CAPP – Scalp Psoriasis	✔	✔
CAPP – Nail Psoriasis	✔	✔
CAPP – Inverse	✔	✔
CAPP – Palm/Sole	✔	✔
CAPP – Genital	✔	✔
Work Productivity		
Work Productivity and Activity Impairment (WPAI)		✔
Fatigue		
Functional Assessment of Chronic Illness Therapy (FACIT-4)		✔
Physical Activity		
International Physical Activity Questionnaire (IPAQ)		✔

The following instruments were selected for COPPAR: For determining and recording PsA, CASPAR criteria were applied to potential subjects by a rheumatologist [15]. PsO diagnosis was based on expert dermatologist evaluation and/or a skin biopsy [14, 16]. The 66/68 tender/swollen joint count was conducted by a Rheumatologist for patients with psoriatic arthritis, as a peripheral joint assessment [19]. Enthesitis was assessed using the LEEDS Enthesitis Index (LEI) [20]. Involvement of dactylitis in the hands and/or feet was documented as absent or present (dactylitis count). Axial involvement (past and current) was documented by the physician and was then assessed by the patient using the Bath Ankylosing Spondylitis Disease Activity Index (BASDAI) [21]. To record quantity and severity of skin lesions we used the Psoriasis Areas Severity Index (PASI) [22], Body Surface Area (BSA) [23, 24], and Physician Static Global Assessment (sPGA) [25, 26].

The clinical outcomes measures included both patient and provider derived scores. In patients with PsA, the Multi-Dimensional Health Assessment Questionnaire (MDHAQ) was used to assess pain and functioning [27]. For evaluating the overall health related quality of life we used the European Quality of Life (EuroQoL) instrument [28]. For a more disease specific assessment of quality of life the Dermatology Life Quality Index (DLQI) was used [29, 30], and for patients with PsA the Psoriatic Arthritis Quality of Life (PsAQoL) questionnaire was additionally recorded [31].

We used a validated patient-derived novel PsO outcomes measure, the Comprehensive Assessment of the Psoriasis Patient (CAPP) [32]. CAPP measures plaque, nail, scalp, inverse, genital and palmo-plantar psoriasis with an equally weighted (1 through 5) physician objective measure and patient-derived, patient-reported outcome measures (visual analog scales).

Visual Analog Scales (VAS) were used to determine and document patient-perceived pain (with or without arthritis), Patient Global Assessment (PGA), and Physician Global Assessment (PGA) [19, 33]. We used patient reported assessments of work productivity, fatigue, physical activity and mental health. The Work Productivity and Activity Impairment (WPAI) questionnaire was used to assess work productivity [34, 35], the Functional Assessment of Chronic Illness Therapy (FACIT, version 4) was used to assess patient fatigue [36], and the International Physical Activity Questionnaire (IPAQ) was used to determine patients’ physical activity [37, 38].

In addition to these validated instruments, we documented past and current use of medications including topical agents. During follow-up visits any changes in medication were recorded, including the reason for the change, be it payment issues/insurance problems, treatment failure or adverse reactions. The questionnaires also assess, socio-demographic status, first diagnosis and a family history of psoriasis (with and/or without arthritis), general health features, life style factors, and health care utilization [39, 40].

### Registry procedures

**Fig. 1:**
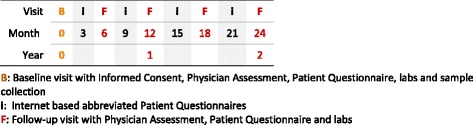
Typical follow-up schedule for patients in the COPPAR registry. : Baseline visit with Informed Consent, Physician Assessment, Patient Questionnaire, labs and sample collection. **i**: Internet based abbreviated Patient Questionnaires. : Follow-up visit with Physician Assessment, Patient Questionnaire and labs

After patients have been identified using the electronic medical records system, they are prioritized and invited to visit the SARM clinic of the Brigham and Women’s Hospital. Such visits may coincide with dermatology/rheumatology visits or may be scheduled in addition. Prior to their scheduled clinic visits a research assistant prepares the administrative paperwork (informed consent, bio-specimen consent, information materials, prospective visit schedule) and physician and patient questionnaires. Upon informed consent, patients undergo a full examination by their treating physician, fill in the patient questionnaire, have blood drawn, and receive an in person follow-up visit schedule for every six months with an internet based questionnaire follow-up sent at three month time points between in person visits (Fig. 1). Patients are emailed a link to the internet based patient questionnaire generated with REDCap (Research Electronic Data Capture): an encrypted, internet-based, electronic data capture tool in line with HIPPA regulations and developed for data capture in research studies [41].

We will collect the following biospecimens annually and then at other specified time points during follow-up: plasma, serum, DNA, RNA, and peripheral blood mononuclear cells (Table 2). These samples will be distributed in aliquots and stored for future use on biomarker studies. In addition, all subjects with PsO will be asked for an optional skin biopsy of affected skin. We expect that a meaningful proportion of subjects will consent for skin biopsy. Joint fluid is retained for COPPAR if arthrocentesis is performed for otherwise clinically indicated interventions. Results from ultrasound or other imaging studies are available through the EMR system.

**Table 2:** Bio-specimens Collection Plan for the COPPAR registry

Blood Collection Schedule	Baseline	12 months	24 months
Plasma Blood Monocyte Count	✔	✔	✔
Plasma	✔	✔	✔
Serum	✔	✔	✔
DNA	✔		
RNA	✔	✔	✔
Skin Biopsy	✔	✔	✔

To ensure high quality data in the registry, the following procedures are followed. Patient questionnaires are reviewed by trained research staff with patients still present to clarify responses. As well, scanners can read all questionnaires reducing human data entry error.

### Analyses

The COPPAR registry data will be analyzed to answer questions in four priority areas. First, we will examine a longitudinal disease states and progression in patients with PsO and PsA. This includes stratification by baseline disease phenotype and if available biomarkers and genotypes. Second, care patterns over time will be described, such as health services use, medication use, and use of other medical interventions. This includes comparing individual care patterns regarding their baseline health status and health outcomes after sufficient risk adjustment. Analyses can be stratified by baseline disease phenotype and biomarkers and genotype. Third, we will assess health outcomes of defined disease states and identify how biomarkers predict disease progression. Fourth, the comparative effectiveness of newer immunomodulating medications will be analyzed.

For the preliminary analysis included here, we searched the EMR system of the Brigham and Women’s Hospital using the algorithm described above to identify candidate patients for COPPAR. Patients were stratified according to a PsO and PsA diagnosis. For both patient groups we tabulated key demographics, comorbidities, and treatment characteristics as derived from the EMR system. Using the medical record number, we crosschecked whether bio-specimens of these patients were already available in the Dermatology Biobank [42]. For both patient groups we then tabulated the frequency of existing bio-specimens.

### Data availability

The registry described in the current study can be inspected jointly with the corresponding author on reasonable request. Upon request, data will be shared with qualified investigators after a material transfer agreement is executed.

### Preliminary results

In our search of the Brigham and Women’s Hospital’s EMR system, we identified a total of 2484 candidate patients. Of these, 1694 had PsO and 1028 had PsA; 238 patients of the 1694 PsO patients also qualified as having PsA based on their diagnosis coding, indicating a transition from PsO to PsA. The mean age was 55 years, with 47% being male. Representative for the Greater Boston area, 85% of patients were white, 3% African-American, and 6% Hispanic. 83% of the patients had at least one visit for PsO or PsA in 2014. On average they had three clinic visits at the BWH in a single year. Corresponding to the patients’ age distribution, 17% had diabetes, 38% hyperlipidemia and 45% hypertension. The average BMI was 29.6. At the time of identification, 33% of the patients used prednisone and 10% used NSAIDs, 86% used topical psoriasis medications. Among PsA patients the current or prior use of MTX was 60% and use of TNFi 66%. The use of these medications was lower in PsO patients with MTX by 20% and TNFi use by 20% (Table 3). Among these patients, we identified a total of 1299 biospecimens already available in the Brigham Biobank. Among PsA patients, 26% had a DNA sample available, among PsO patients 11% had DNA available (Table 4).

**Table 3:** Currently Eligible Patient Population for the COPPAR registry^a^

	Psoriasis (*N* = 1694)**	Psoriatic Arthritis (*N* = 1028)**	Total Cohort (*N* = 2484)
	*N (%) or mean (+/−SD)*		
Demographics/Health Services Use			
Age, mean, years	55.5 (± 16.2)	55.9 (± 14.2)	55.5 (± 15.6)
Male sex	788 (46.5)	494 (48.1)	1162 (46.8)
Race			
White	1382 (81.6)	917 (89.2)	2099 (84.5)
Black	61 (3.6)	19 (1.8)	69 (2.8)
Hispanic	127 (7.5)	23 (2.2)	139 (5.6)
Other	124 (7.3)	69 (6.7)	177 (7.1)
Patients with visits in 2014 for PsO/PsA	1404 (82.9)	841 (81.8)	2068 (83.3)
Visits in 2014 for PsO/PsA, mean	3 (± 5)	3 (± 2)	3 (± 4)
Comorbidities			
Diabetes	318 (18.8)	169 (16.4)	420 (16.9)
Hyperlipidemia	740 (43.7)	302 (29.4)	942 (37.9)
Hypertension	835 (49.3)	422 (41.1)	1110 (44.7)
Body mass index, mean	29.5 (± 7.7)	29.8 (± 6.9)	29.6 (± 7.4)
Psoriasis and Psoriatic Arthritis Characteristics			
Prednisone use	470 (27.7)	478 (46.5)	833 (33.5)
Current NSAID use	107 (6.3)	153 (14.9)	248 (10.0)
Current/prior MTX use	335 (19.8)	616 (59.9)	801 (32.2)
Current/prior TNFi	350 (20.7)	675 (65.7)	858 (34.5)
Current/prior topical PsO	1625 (95.9)	728 (70.8)	2123 (85.5)

^a^ Based on electronic medical record review

** At least three diagnoses of PsO (ICD-9-CM code 696.1 or ICD-10 code L40.0) or at least three diagnoses of PsA (ICD-9-CM code 696.0 or ICD-10 code L40.5)

**Table 4:** Current Bio-specimen repository for the COPPAR registry

Blood Collection Schedule	Psoriasis		Psoriatic Arthritis	
	N	%	N	%
Plasma Blood Monocyte Count	153	9.0%	106	6.3%
Plasma	190	11.2%	220	13.0%
Serum	0	0.0%	33	1.9%
DNA	187	11.0%	266	15.7%
RNA	91	5.4%	48	2.8%
Skin Biopsy	2	0.1%	3	0.2%

## Discussion

The aim of the COPPAR registry of patients with PsO and/or PsA was to develop a comprehensive longitudinal data asset that allows researchers to determine predictors of PsA among patients initially presenting with PsO; to identify predictors of treatment response and failure and characterize treatment transitions; to quantify health services utilization and quality of life of patients with PsO and PsA; to assess relevant subgroups of patients with specific phenotypes; and to determine the effectiveness and safety of new treatments.

The conception and methodology of COPPAR follows the successful implementation of BRASS (Brigham Rheumatoid Arthritis Studies) that exists since 2003, and is frequently used for research on disease progression and the safety and effectiveness of medical treatment [42–45].

Specific advantages of COPPAR are that it leverages an infrastructure already developed for BRASS, a rheumatoid arthritis registry, that it is a single center cohort with large clinical caseload of interconnected dedicated dermatology and rheumatology practices, that it has access to electronic medical record and an existing bio-repository, that patients receive various pharmacologic strategies allowing for non-randomized comparative effectiveness and biomarker studies, and that patients can be re-contacted by mail or electronically for additional prospective studies (trials, additional biomarkers). The participating physicians and investigators have strong clinical and academic track records in all relevant areas, such as clinical care of PsO and PsA, biobanking, comparative effectiveness research, pharmacoepidemiology, and patient-reported outcomes measurement. The registry team integrates strong expertise in skin and joint diseases into a single registry.

Recently established, the COPPAR registry is actively recruiting patients with PsO and PsA starting May 2017 with the goal to quickly complete a base cohort of 1000 PsO and PsA patients each. These patients will be followed long-term and additional patients will join over time.

## Abbreviations

BASDAI: Bath Ankylosing Spondylitis Disease Activity Index
BSA: Body Surface Area
CASPAR: Classification criteria for psoriatic arthritis
COPPAR: Cohort for Psoriasis and Psoriatic Arthritis Registry
EMR: Electronic medical record
FACIT: Functional Assessment of Chronic Illness Therapy
IPAQ: International Physical Activity Questionnaire
LEI: LEEDS Enthesitis Index
PASI: Psoriasis Areas Severity Index
PBMCs: Peripheral blood mononuclear cells
PGA: Patient Global Assessment, and Physician Global Assessment
PsA: Psoriatic arthritis
PsO: Psoriasis
REDCap: Research Electronic Data Capture
SARM: Skin and Related Musculoskeletal Diseases
sPGA: Physician Static Global Assessment
TNFi: Tumor necrosis factor alpha inhibitor
VAS: Visual analog scales
WPAI: Work Productivity and Activity Impairment

## References

[1] Gliklich RE, Dreyer N (2010). Registries for evaluating patient outcomes: a User’s guide.

[2] Augustin M, Spehr C, Radtke MA, et al. German psoriasis registry PsoBest: objectives, methodology and baseline data. *Journal der Deutschen Dermatologischen Gesellschaft = Journal of the German Society of Dermatology : JDDG.* Jan 2014;12(1):48–57. 10.1111/ddg.12233 24393314

[3] Lofvendahl S, Petersson IF, Theander E, Svensson A, Zhou C, Steen Carlsson K. Incremental Costs for Psoriasis and Psoriatic Arthritis in a Population-based Cohort in Southern Sweden: Is It All Psoriasis-attributable Morbidity? *The Journal of rheumatology.* Jan 15 2016. 10.3899/jrheum.150406 26773111

[4] Khraishi M, MacDonald D, Rampakakis E, Vaillancourt J, Sampalis JS (2011). Prevalence of patient-reported comorbidities in early and established psoriatic arthritis cohorts. Clin Rheumatol.

[5] Carneiro JN, Paula AP, Martins GA (2012). Psoriatic arthritis in patients with psoriasis: evaluation of clinical and epidemiological features in 133 patients followed at the University Hospital of Brasilia. An Bras Dermatol.

[6] Sarzi-Puttini P, Varisco V, Ditto MC, Benucci M, Atzeni F (2015). Psoriatic arthritis registries. The Journal of rheumatology Supplement Nov.

[7] Ogdie A, Weiss P (2015). The epidemiology of psoriatic arthritis. Rheum Dis Clin N Am.

[8] Liu JT, Yeh HM, Liu SY, Chen KT. Psoriatic arthritis: Epidemiology, diagnosis, and treatment. *World journal of orthopedics.* Sep 18 2014;5(4):537–543. 10.5312/wjo.v5.i4.537 PMC4133459 25232529

[9] Ibrahim G, Waxman R, Helliwell PS. The prevalence of psoriatic arthritis in people with psoriasis. *Arthritis and rheumatism.* Oct 15 2009;61(10):1373–1378. 10.1002/art.24608 19790120

[10] Velez NF, Wei-Passanese EX, Husni ME, Mody EA, Qureshi AA (2012). Management of psoriasis and psoriatic arthritis in a combined dermatology and rheumatology clinic. Arch Dermatol Res.

[11] Eder L, Gladman DD (2014). Predictors for clinical outcome in psoriatic arthritis - what have we learned from cohort studies?. Expert Rev Clin Immunol.

[12] Azevedo VF, Buiar PG (2013). Risk factors and predictors of psoriatic arthritis in patients with psoriasis. An Bras Dermatol.

[13] Lofvendahl S, Theander E, Svensson A, Carlsson KS, Englund M, Petersson IF (2014). Validity of diagnostic codes and prevalence of physician-diagnosed psoriasis and psoriatic arthritis in southern Sweden--a population-based register study. PLoS One.

[14] Asgari MM, Wu JJ, Gelfand JM (2013). Validity of diagnostic codes and prevalence of psoriasis and psoriatic arthritis in a managed care population, 1996-2009. Pharmacoepidemiol Drug Saf.

[15] Taylor W, Gladman D, Helliwell P (2006). Classification criteria for psoriatic arthritis: development of new criteria from a large international study. Arthritis Rheum.

[16] Marks R, Barton SP, Shuttleworth D, Finlay AY (1989). Assessment of disease progress in psoriasis. Arch Dermatol.

[17] Radloff L (1977). The CES-D scale: a self-report de- pression scale for research in the general population. Appl Psychol Meas.

[18] Mease PJ. Measures of psoriatic arthritis: Tender and Swollen Joint Assessment, Psoriasis Area and Severity Index (PASI), Nail Psoriasis Severity Index (NAPSI), Modified Nail Psoriasis Severity Index (mNAPSI), Mander/Newcastle Enthesitis Index (MEI), Leeds Enthesitis Index (LEI), Spondyloarthritis Research Consortium of Canada (SPARCC), Maastricht Ankylosing Spondylitis Enthesis Score (MASES), Leeds Dactylitis Index (LDI), Patient Global for Psoriatic Arthritis, Dermatology Life Quality Index (DLQI), Psoriatic Arthritis Quality of Life (PsAQOL), Functional Assessment of Chronic Illness Therapy-Fatigue (FACIT-F), Psoriatic Arthritis Response Criteria (PsARC), Psoriatic Arthritis Joint Activity Index (PsAJAI), Disease Activity in Psoriatic Arthritis (DAPSA), and Composite Psoriatic Disease Activity Index (CPDAI). *Arthritis care & research.* Nov 2011;63 Suppl 11:S64–85. 10.1002/acr.20577 22588772

[19] Chandran V, Gottlieb A, Cook RJ, et al. International multicenter psoriasis and psoriatic arthritis reliability trial for the assessment of skin, joints, nails, and dactylitis. *Arthritis and rheumatism.* Sep 15 2009;61(9):1235–1242. 10.1002/art.24562 19714610

[20] Healy PJ, Helliwell PS. Measuring clinical enthesitis in psoriatic arthritis: assessment of existing measures and development of an instrument specific to psoriatic arthritis. *Arthritis and rheumatism.* May 15 2008;59(5):686–691. 10.1002/art.23568 18438903

[21] Taylor WJ, Harrison AA. Could the Bath Ankylosing Spondylitis Disease Activity Index (BASDAI) be a valid measure of disease activity in patients with psoriatic arthritis? *Arthritis and rheumatism.* Jun 15 2004;51(3):311–315. 10.1002/art.20421 15188312

[22] Langley RG, Ellis CN (2004). Evaluating psoriasis with psoriasis area and severity index, psoriasis global assessment, and lattice system Physician's global assessment. J Am Acad Dermatol.

[23] Long CC, Finlay AY, Averill RW (1992). The rule of hand: 4 hand areas = 2 FTU = 1 g. Arch Dermatol.

[24] Ashcroft DM, Wan Po AL, Williams HC, Griffiths CE (1999). Clinical measures of disease severity and outcome in psoriasis: a critical appraisal of their quality. Br J Dermatol.

[25] Feldman SR, Krueger GG. Psoriasis assessment tools in clinical trials. *Annals of the rheumatic diseases.* Mar 2005;64 Suppl 2:ii65–68; discussion ii69–73. 10.1136/ard.2004.031237 PMC1766877 15708941

[26] Chow C, Simpson MJ, Luger TA, Chubb H, Ellis CN (2015). Comparison of three methods for measuring psoriasis severity in clinical studies (part 1 of 2): change during therapy in psoriasis area and severity index, static Physician's global assessment and lattice system Physician's global assessment. Journal of the European Academy of Dermatology and Venereology : JEADV.

[27] Husted J, Gladman DD, Farewell VT, Long JA (1996). Validation of the revised and expanded version of the arthritis impact measurement scales for patients with psoriatic arthritis. J Rheumatol.

[28] Yang Y, Brazier J, Longworth L (2015). EQ-5D in skin conditions: an assessment of validity and responsiveness. The European journal of health economics : HEPAC : health economics in prevention and care.

[29] Finlay AY, Khan GK (1994). Dermatology life quality index (DLQI)--a simple practical measure for routine clinical use. Clin Exp Dermatol.

[30] Nichol MB, Margolies JE, Lippa E, Rowe M, Quell J (1996). The application of multiple quality-of-life instruments in individuals with mild-to-moderate psoriasis. PharmacoEconomics.

[31] Healy PJ, Helliwell PS (2008). Psoriatic arthritis quality of life instrument: an assessment of sensitivity and response to change. J Rheumatol.

[32] Patel M, Liu SW, Qureshi A, Merola JF (2014). The Brigham scalp nail inverse palmoplantar psoriasis composite index (B-SNIPI): a novel index to measure all non-plaque psoriasis subsets. J Rheumatol.

[33] Cauli A, Gladman DD, Mathieu A (2011). Patient global assessment in psoriatic arthritis: a multicenter GRAPPA and OMERACT study. J Rheumatol.

[34] Zhang W, Bansback N, Boonen A, Young A, Singh A, Anis AH (2010). Validity of the work productivity and activity impairment questionnaire--general health version in patients with rheumatoid arthritis. Arthritis research & therapy.

[35] Tillett W (2012). De-Vries C, McHugh NJ. Work disability in psoriatic arthritis: a systematic review. Rheumatology.

[36] Chandran V, Bhella S, Schentag C, Gladman DD (2007). Functional assessment of chronic illness therapy-fatigue scale is valid in patients with psoriatic arthritis. Ann Rheum Dis.

[37] Hagstromer M, Oja P, Sjostrom M (2006). The international physical activity questionnaire (IPAQ): a study of concurrent and construct validity. Public Health Nutr.

[38] Torres T, Alexandre JM, Mendonca D, Vasconcelos C, Silva BM, Selores M (2014). Levels of physical activity in patients with severe psoriasis: a cross-sectional questionnaire study. Am J Clin Dermatol.

[39] Iannaccone CK, Lee YC, Cui J (2011). Using genetic and clinical data to understand response to disease-modifying anti-rheumatic drug therapy: data from the Brigham and Women's Hospital rheumatoid arthritis sequential study. Rheumatology.

[40] Bykerk VP, Shadick N, Frits M (2014). Flares in rheumatoid arthritis: frequency and management. A report from the BRASS registry. J Rheumatol.

[41] Pittman CA, Miranpuri AS (2015). Neurosurgery clinical registry data collection utilizing informatics for integrating biology and the bedside and electronic health records at the University of Rochester. Neurosurg Focus.

[42] Centola M, Cavet G, Shen Y (2013). Development of a multi-biomarker disease activity test for rheumatoid arthritis. PLoS One.

[43] Lee YC, Frits ML, Iannaccone CK, et al. Subgrouping of patients with rheumatoid arthritis based on pain, fatigue, inflammation, and psychosocial factors. Arthritis & rheumatology Aug 2014;66(8):2006–2014. 10.1002/art.38682 PMC4188637 24782222

[44] Cui J, Taylor KE, Lee YC (2014). The influence of polygenic risk scores on heritability of anti-CCP level in RA. Genes Immun.

[45] Lillegraven S, Paynter N, Prince FH (2013). Performance of matrix-based risk models for rapid radiographic progression in a cohort of patients with established rheumatoid arthritis. Arthritis care & research.

